# Perceptions of radiation safety training among interventionalists in South Africa

**DOI:** 10.5830/CVJA-2017-028

**Published:** 2017

**Authors:** André Rose, William Ian Duncombe Rae

**Affiliations:** Department of Community Health, University of the Free State, Bloemfontein, South Africa; Department of Medical Physics, University of the Free State, Bloemfontein, South Africa

**Keywords:** radiation safety training, interventionalists’ training, radiation awareness, occupational radiation safety, cardiology training

## Abstract

Exposure to ionising radiation may have deterministic and stochastic health effects, which include skin changes, chromosomal aberrations, cataracts and carcinomas. Formalised training in radiation safety and protection improves knowledge on the subject and facilitates greater compliance in safety practices. This qualitative study included 54 interventionalists (adult and paediatric cardiologists, and interventional radiologists). The participants were purposively selected and interviewed to explore their perceptions about radiation safety. A thematic analysis of the transcripts was done using a deductive and inductive approach. Findings showed participating cardiologists had less knowledge about radiation safety than participating radiologists. Cardiologists reported little or no formal training on radiation safety and did not display a culture of radiation safety. There was no consensus on how the training gap should be addressed. There is a perceived need to change and enhance the radiation safety culture among interventionists, and the participants proffered some ideas. These included the need for re-curricularisation of cardiologists’ training to create awareness of radiation safety practices.

## Introduction

Continuous improvements are taking place in radiological imaging technology, with an accompaning reduction in radiation exposures required for imaging.^1^ There has however also been an increase in patient load, and fluoroscopic procedures are becoming more complex and taking longer to perform.^2^, ^3^ This consequently increases radiation exposure to operators.

Evidence is mounting that even at low-dose exposure, there are important biological consequences.^4^ Ionising radiation can produce detrimental biological effects, which include acute and chronic skin effects, chromosomal abnormalities, various carcinomas and cataracts.^5^-^7^ The effects of radiation exposure may be deterministic or stochastic.^8^ It is therefore imperative that health professionals working with ionising radiation are adequately informed and trained on the dangers associated with using this modality, so they can protect themselves better.^9^

Adequate understanding of the effects of occupational radiation exposure and vigilant radiation safety practices among interventionists are essential to protect the health of this group of healthcare professionals. It is concerning that interventional cardiologists need to make decisions about radiation use for their patients and protection for themselves with the level of training they receive in radiobiology and radiation physics.^9^ The required knowledge level may be effectively achieved by incorporating changes in their training curriculum and in on-going continued medical education (CME) programmes, as is evidenced by radiology training programmes.^1^

Training and formal lectures targeted at developing a culture of radiation safety are crucial to developing a culture of radiation safety.^10^ Radiation physics and radiobiology is part of the curriculum for radiology registrars in South Africa. They are examined on these topics in their Part I examination, but have no subsequent examination on these topics.^11^

Rehani argues that the intensity of radiation used by interventional cardiologists is no less than that used by interventional radiologists and for this reason, the two disciplines should have similar training in radiobiology and radiation physics.12 This is however not practical at present in South Africa and requires an alternative approach to improving radiation safety knowledge, awareness and practice in non-radiologist clinicians.^12^

Interventionalists are highly skilled doctors. In South Africa, there is a dearth of skilled medical personnel and an even greater shortage of highly skilled interventionalists. The demand for this skill is not being met by the output of subspecialists qualifying.^13^ It is therefore crucial to protect the health of those already in service and those who will enter the field. Adequate training is not just about developing skills acumen, but also instilling vigilant radiation safety practices, and this can be entrenched through the formal training curriculum. Influencing changes in a curriculum is challenged by various factors, such as prevailing perceptions from the fraternity.

The aim of this article was to report on the perception of South African interventionalists on radiation education and safety training.

## Methods

This was a qualitative study in which we conducted group and in-depth interviews. Qualitative research aims to capture the specific voice of the participants on this topic by producing rich insights into the experiences, values and understanding of participants on the matter.^14^

Thirty individual interviews were conducted and six group interviews with between two and six participants were facilitated. Table 1 provides a detailed description of the participants, who were predominantly male (61%). The study population consisted of adult interventional cardiologists, paediatric interventional cardiologists and interventional radiologists, and are referred to collectively as interventionalists. Adult and paediatric cardiologists are collectively referred to as cardiologists, unless otherwise specified.

**Table 1:** Demographic characteristics of the participants (n = 54)

*Parameters*	*Number (%)*
Gender	
Male	33 (61.1)
Female	21 (38.9)
Median age (years)	41 (IQR 35–55)
Median years worked	6.5 (IQR 2–20)
Categories of professionals	
All interventionalists	54
Radiologists	16 (29.6)
Radiology registrars	13 (24.1)
Adult cardiologists	10 (18.5)
Adult cardiology fellows	6 (11.1)
Paediatric cardiologists	7 (13.0)
Paediatric cardiology fellows	2 (3.7)
Sector worked				
Public only	29 (53.7)
Private only	9 (13.0)
9 (13.0)	18 (33.3)
Levels of training	
In training^1^	21 (38.9)
Junior professionals^2^	4 (7.4)
Mid-level professionals^3^	12 (22.2)
Senior professionals^4^	11 (20.4)
Heads of departments	6 (11.1)
City worked in	
Johannesburg	17 (31.5)
Bloemfontein	13 (24.1)
Cape Town	9 (16.7)
Pretoria	5 (9.3)
Other^5^	7 (12.9)
Outside of South Africa6	3 (5.5)

IQR, interquartile range; ^1^cardiology fellows and radiology registrars; ^2^less than five years post qualifying; ^3^five to 15 years post qualifying; ^4^more than 15 years post qualifying; ^5^Durban, Kimberley, Mthatha, Pietermaritzburg; ^6^Australia, New Zealand, United Kingdom.

The participants were purposively selected because they could contribute to an understanding of the perception of South African interventionalists on radiation education and safety training.^15^,^16^ We used targeted sampling in this study,^17^,^18^ and approached specific informants, such as the heads of departments, to participate in the study.

The purposive selection also ensured that participants represented the opinions of people with a wide range of demographic characteristics, including those from different regions, levels of training, professions, and sectors where they worked, as shown in Table 1. We therefore attempted to include the full range of people involved, to get a clear impression of the overall feeling within South Africa.

We commenced the qualitative data collection in May 2015 and ended in July 2016 when we determined that data saturation had been reached and there was a representative spread of all categories of professionals. Data were collected at several conferences and workshops using an interview schedule. Participants were asked what they thought the radiation safety training requirements for their respective disciplines were, whether the requirements matched their expectations, and if there was room for improvement, how a change could be executed.

The study was approved by the Human Research Ethics Committee of the Faculty of Health Sciences of the University of the Free State (ECUFS 44/2015). Written informed consent was obtained from all participants. In the discussion groups, the participants were asked not to divulge their responses outside the group.

## Statistical analysis

Thematic analysis using a deductive and inductive approach was used.^19^,^20^ The interviews were audio-recorded and transcribed verbatim. We then checked the transcripts against the audio recordings for accuracy. Data included the researcher’s field notes.

Data were analysed as we received it. We used Braun and Clarke’s steps in the analysis process.^21^ The researchers independently read the transcripts and coded the data. The codes were organised into categories and the categories were grouped into themes. We discussed the interpretations that emerged. We debated the themes and then reached consensus on the findings. This article explores only the theme of radiation safety, training and education.

## Results

The main themes that were formed included: ‘knowledge and awareness of radiation effects’, ‘education and training in radiation safety’, and ‘the role of senior professionals in fostering a culture of education and training’. In the quotes below (AC) refers to adult cardiologists, (PC) to paediatric cardiologists and (R) to radiologists. There was no difference between men and women in how they responded to the training they received.

## Knowledge and awareness of radiation effects

Radiologists generally had a well-informed opinion about how ionising radiation worked and the effects it could have on their health and the health of their patients. As one radiologist reported:

‘I don’t think there is any theory that we’re missing out on [in training] if you do the proper course work for your primary exams. I think that covers everything that’s necessary’ (R).

Radiologists often spoke confidently about how radiation affected health and consistently described the consequences as ‘stochastic and deterministic effects’ (R). They displayed a familiarity with the literature on the topic.

This contrasted with the cardiologists whose understanding resonated with what you would expect from a non-radiologist doctor. A paediatric cardiology fellow reflected on the effects of ionising radiation on her health as follows:

‘I haven’t thought about it [laughs] to be completely honest. We go there [the cath lab] each week and we have our little [dosimeter] badges. We don’t really think about what’s happening’ (PC). A radiologist from New Zealand corroborated this view stating:

‘It is assumed that the doctors understand about radiation, but this isn’t the case’ (R).

## Education and training in radiation safety

There was a distinct difference between cardiologists and radiologists in their training in radiobiology and radiation physics. The cardiologists receive very little or no formal training in these subjects while radiologists have it as part of their coretraining curriculum.

‘I think as postgraduates they [radiology registrars] get enough training on radiation safety. I would like to see it [radiation safety] as part of every imaging congress for the staff because it is often neglected. But if you have an imaging congress, that must be part of it; to remind all the people at the congress about radiation protection. I think that will go a far way already in reminding them about safety measures and radiation protection. And then in our normal academic programme to just make sure that it receives enough attention’ (R).

‘Perhaps [there should be] a short course on the amount of exposure that there is you get in relation to how much work you do. You know, a couple of lectures or a lecture on that. Uhmm and to ... implement that into the [cath] lab. But that’s what I think should be done; I had no training about radiation whatsoever, not … in any way’ (AC).

‘Cause they [cardiologists] didn’t do physics, they haven’t done like physics, like part of our training is physics and [it is in] the exam, it is not part of their training. I don’t know if they actually are aware of it [the effects of radiation]’ (R) reflecting on cardiology training in radiation safety.

‘I think it [radiation physics] should be highlighted as something [that should] at least be done at the first year’ (R), in response to training in radiation safety for doctors using ionising radiation as a modality.

Junior and recently qualified cardiologists expressed concern that they were using a modality that could have dire consequences to their long-term health, but were not being trained in how to safely use radiation.

‘We don’t really have training, it is just like we do selfstudy for physics’ (AC) fellow.

The paediatric cardiology heads of departments that participated in the study unanimously expressed the opinion that it was an important but neglected aspect of the content of their training programmes. They generally held the view that more could and should be done to improve the training and awareness on the topic. They however expressed the concern that their departments were not necessarily equipped to do such training and that other departments such as medical physics should assist with this training. (After the interview with the PC quoted below, a question on radiation safety was asked in the CMSA examinations for paediatric cardiology in April 2016.)

‘I’ve been an examiner in paediatric cardiology for a while. For over 10 years I haven’t seen a question about it [radiation safety]. So, it’s not of importance and nobody discusses it. So, there should be training and it should be in the curriculum…’ (PC).

‘It [radiation safety] is something that we’ve never discussed or ever brought up in a meeting until you came along actually. How do we incorporate that when we train our fellows? I don’t remember ever been told anything by my consultants with regards to radiation safety for myself when I was being trained. Maybe we can add some training because at the moment there isn’t any. There’s no training!’ (PC).

Adult cardiology heads of department (HOD) were divergent on their views. They recognised that radiation safety was important and lacking in their programmes, but did not think that the current training programme needed re-evaluation. They were concerned that the volume of work was already too much for the cardiology fellows.

‘Ja, it’s hard to uhhh, cardiology is vast on its own. Adding a section on radiation is asking a bit much. But I think uhhmm, in the syllabus, that we give, you know there is a syllabus, a cardiologist syllabus for the trainees. Somewhere in that syllabus it should emphasise the fact or some knowledge should be given around radiation and the issues about radiation’ (C) HOD.

Participants reflected that education and training was however not a once-off exercise and that frequent and constant reinforcing was needed.

‘I do think education does have a very important role if one is making people aware. I would hope that you would then gradually improve their performance in the lab but it is something I think needs reinforcing regularly because I’ve seen very experienced operators still behaving badly in the cath lab. I’ve seen it a huge amount. And so, I think we just have to keep reinforcing good practice and keep educating them’ (R) UK.

## The role of senior professionals in fostering a culture of education and training

At one training institution, the head of adult cardiology was very dismissive of the topic. This HOD was reluctant to participate in the study, stating that a more junior cardiologist should be interviewed. Despite explaining the nature of the study and stating that we were interested in hearing his/her voice as HOD on training in radiation safety, the HOD was still not interested in participating in the study. This created the impression that the HODs of some training units were not interested in the topic. This dismissive attitude towards radiation safety was also recognised by other participants in the study: ‘Yes, yes they [heads of departments] are shocking yes. No,no, no that’s exactly true and that’s certainly true and I can promise you that is not just in South Africa. That will be all over ja’ (R) UK.

## Discussion

The increasing utilisation of ionising radiation for diagnostic, therapeutic and interventional procedures necessitates great vigilance in using the modality. This strengthens the case for interventionalists to be adequately trained in the use of radiation. Improving knowledge on the effects of ionising radiation on the health of patients and operators requires improved access to training and education on the topic. The literature consistently cites that formalising radiation safety and training in the curriculum is essential for improving and maintaining radiation safety practices for interventionalists.^1^,^22^

In a study by Sadighm et al., they found an increased awareness about radiation among radiology residents compared to non-radiology residents.^9^ Even though we had not quantified our findings, we interpreted that radiologists were generally more knowledgeable on the effects of radiation and safety precautions compared to cardiologists. We postulate that this is because of the formalisation of radiobiology and radiation physics in their training curriculum. It is concerning that this discrepancy exists, as cardiologists are exposed to similar radiation workloads to radiologists and the dose exposure is likely to increase as the complexity of cardiology procedures increases.^12^

An effective way to improve the knowledge of radiation safety is to provide opportunities for education and training in the subject during specialisation. Limacher et al. argues that the best way to ensure adequate training in radiation safety is to formalise it in the curriculum.^5^ Radiobiology and radiation physics is mandatory for radiologists in South Africa and is a formal part of their training programme.^11^ This is not the case for cardiologists in South Africa (personal communication). Szarmach et al. state that radiation safety can only be addressed by educating all healthcare professionals, irrespective of their position, and that they need to be trained ‘thoroughly and systematically’.^23^ Reinforcing radiation safety messages and training optimises radiation safety.^24^

The Colleges of Medicine curriculum prescribes radiation physics and radiobiology as learning outcomes for both radiologists and cardiologists.^11^ From the interviews and from personal discussions with interventionalists involved with training specialists, it emerged that there was no standardised teaching of radiation safety at the various training institutions. Instruction in the topic ranged from in-house teaching, registrars or clinical fellows attending short courses, or self-learning on the topic. Uniformity in content and instruction will facilitate that interventionalists are adequately trained in this area and that radiation safety is reinforced across South Africa.^1^,^23^,^24^

It is inconsistent, and hence ineffective, if the leadership of a clinical unit does not actively promote radiation safety and training but expects junior staff to adhere to these principles.^25^ The attitude of the HOD of a unit is key to developing a culture of radiation education and training. Radiation safety as a priority will not permeate the department if those at the helm are not recognising it as a priority and championing the cause.

The views expressed by South African interventionalists were corroborated by at least three international interventionalists. The views of these international doctors are included to illustrate that the experience and challenges of training cardiologists and radiologists in radiation safety are not unique to South Africa.

Developing, strengthening and sustaining a radiation education and training culture in South Africa among interventionalists will require changes in their formal training and deliberate inclusion in their CME programmes. Education is crucial to establishing a radiation safety culture and will require buy-in at all levels.

## Limitations of the study

The participants were purposively sampled and the findings are not generalisable to the whole population of South African interventionists. The participants however reflect the population of interest and the findings may be transferable in similar settings. The findings highlight that radiation safety is an important aspect of training and that it is imperative to adequately train interventionalists in this field. Further research is needed to better understand this issue and how to incorporate it into interventionalists’ training programmes.

## Conclusion

Radiobiology and radiation physics is formalised in the training curriculum for radiologists, resulting in greater awareness about radiation dangers and greater vigilance in radiation safety practice. There is a paucity of knowledge about radiation safety practices among cardiologists in South Africa, and cardiologists need to be empowered to make more informed decisions about using ionising radiation, in order to protect themselves and their patients. This can be achieved by including it in their formal training curriculum and raising the expected outcomes to that of radiologists.

## References

[1] Le Heron J, Padovani R, Smith I, Czarwinski R (2010). Radiation protection of medical staff.. Eur J Radiol.

[2] Bhargavan M (2008). Trends in the utilization of medical procedures that use ionizing radiation.. Heal Phys.

[3] Smilowitz NR, Balter S, Weisz G (2013). Occupational hazards of interventional cardiology.. Cardiovasc Revasc Med.

[4] Shore RE, Neriishi K, Nakashima E (2010). Epidemiological studies of cataract risk at low to moderate radiation doses: (not) seeing is believing.. Radiat Res.

[5] Limacher MC, Douglas PS, Germano G, laskey WK, Lindsay BD, Mcketty MH (1998). Radiation Safety in the practice of cardiology.. J Am Coll Cardiol.

[6] Veneri L, Rossi F, Botto N, Andreassi MG, Salcone N, Emad A (2009). Cancer risk from professional exposure in staff working in cardiac catheterization laboratory: insights from the National Research Council’s Biological Effects of Ionizing Radiation VII Report.. AM Hear J.

[7] Klein LW, Miller DL, Balter S, Laskey W, Haines D, Norbash A (2009). Occupational health hazards in the interventional laboratory : time for a safer environment.. Int J Vasc Interv Radiol.

[8] Navarro VCC, Navarro MVT, Maia AF (2013). Assessments of medical exposuresduring interventional radiology procedures.. Radiat Prot Dosim.

[9] Sadigh G, Khan R, Kassin MT, Applegate KE (2014). Radiation safety knowledge and perceptions among residents: A potential improvement opportunity for graduate medical education in the United States.. Acad Radiol.

[10] Cole P, Hallard R, Broughton J, Coates R, Croft J, Davies K (2014). Developing the radiation protection safety culture in the UK.. J Radiol Prot.

[12] Rehani MM (2007). Training of interventional cardiologists in radiation protection – the IAEA ’s initiatives.. Int J Card.

[13] Sliwa K, Zühlke L, Kleinloog R, Doubell A, Ebrahim I, Essop M (2016). Cardiology–cardiothoracic subspeciality training in South Africa: a position paper of the South Africa Heart Association.. Cardiovasc J Afr.

[14] Sofaer S (2002). Qualitative research methods.. Int J Qual Heal Care.

[15] Morgan DL (2002). Focus group interviewing.. Handb Interview Res Context Method.

[16] Wilkinson S (1998). Focus group methodology: a review.. Int J Soc Res Methodol.

[17] Marshall MN (1996). pling for qualitative research Sample size.. Fam Pract.

[18] Noy C (2008). Sampling knowledge: the hermeneutics of snowball sampling in qualitative research.. Int J Soc Res Methodol.

[19] Vaismoradi M, Turunen H, Bondas T (2013). Content analysis and thematic analysis: Implications for conducting a qualitative descriptive study.. Nurs Heal Sci.

[20] Braun V, Clarke V (2014). What can ‘thematic analysis’ offer health and wellbeing researchers?. Int J Qual Stud Health Well-being.

[21] Braun V, Clarke V (2006). Using thematic analysis in psychology.. Qual Res Psychol.

[22] Lynskey GE, Powell DK, Dixon RG, Silberzweig JE (2013). Radiation protection in interventional radiology: survey results of attitudes and use.. J Vasc Interv Radiol.

[23] Szarmch A, Piskunowicz M, Świętoń D, Muc A, Mockałło G, Dzierżanowski J (2015). adiation safety awareness among medical staff.. Pol J Radiol.

[24] Sheyn DD, Racadio JM, Ying J, Patel MN, Racadio JM, Johnson ND (2008). Efficacy of a radiation safety education initiative in reducing radiation exposure in the pediatric IR suite.. Pediatr Radiol.

[25] Zisook S, Mcquaid JR, Sciolla A, Lanouette N, Calabrese C, Dunn LB (2011). Psychiatric residents’ interest in psychotherapy and training stage: A multi-site survey. Am J Psychother.

